# Preoperative embolization in patients with metastatic spinal cord compression: mandatory or optional?

**DOI:** 10.1186/s12957-017-1118-3

**Published:** 2017-02-14

**Authors:** Chul Gie Hong, Jae Hwan Cho, Dae Chul Suh, Chang Ju Hwang, Dong-Ho Lee, Choon Sung Lee

**Affiliations:** 1 Department of Orthopedic Surgery, CHA Gumi Medical Center, Cha University, Gumi, Korea; 2 Department of Orthopedic Surgery, Asan Medical Center, University of Ulsan College of Medicine, 388-1, PungNap-2-dong, SongPa-gu, Seoul, Korea; 3 Department of Radiology, Asan Medical Center, University of Ulsan College of Medicine, Seoul, Korea

**Keywords:** Embolization, Metastasis, Spine, Cord compression, Hypervascular tumor

## Abstract

**Background:**

The contribution of preoperative embolization in reducing intraoperative blood loss and its clinical importance are unclear. So, we aimed to compare the perioperative clinical outcomes based on whether preoperative embolization was performed and assess the role and safety of preoperative embolization in metastatic spinal cord compression (MSCC) patients.

**Methods:**

We enrolled 52 patients (men, 37; women, 15) who underwent palliative decompression for MSCC. Demographic data, neurologic status, surgery-related data (operation time, estimated blood loss, and transfusion), complications, and survival time were recorded. Patients were categorized based on whether they received preoperative embolization: groups E (embolization) (*n* = 18) and NE (non-embolization) (*n* = 34) and the clinical parameters were compared. Subgroup analysis was performed specifically for cases of hypervascular tumors (23/52, 44%).

**Results:**

The transfusion degree was greater in the NE group (4.6 pints) than in the E group (2.5 pints, *P* = 0.025); the other parameters did not differ between the groups. However, massive bleeding (>2000 mL) was more frequent in the NE group (10/34) than in the E group (0/18, *P* = 0.010). Subgroup analysis indicated that intraoperative blood loss was greater in the NE group (1988 mL) than in the E group (1095 mL, *P* = 0.042) in hypervascular tumor patients. Although massive bleeding was more frequent among hypervascular tumor patients, 3 patients with non-hypervascularized tumors also exhibited massive bleeding (*P* = 0.087).

**Conclusions:**

Intraoperative blood loss and perioperative transfusion can be reduced by preoperative embolization in MSCC patients. Neurologic recovery, operation time, and complications did not differ according to the application of embolization. As preoperative embolization is relatively safe and effective for controlling intraoperative bleeding without any neurologic deterioration, it is highly recommended for hypervascular tumors. Moreover, it may also be effective for non-hypervascular tumors as massive bleeding may be noted in some cases.

## Background

Metastatic spinal cord compression (MSCC) is a critical problem for patients with spinal metastasis. MSCC is reportedly observed in 5–10% of cases with advanced cancer [1]. It is important to diagnose MSCC in the early period to avoid neurologic deficits. However, studies have found that 48% of patients are unable to walk at the time of diagnosis [1]. The treatment of MSCC depends on the primary tumor, neurologic status, progression of limb weakness, tumor burden, performance status, life expectancy, and possibility of other therapeutic options such as radiotherapy [2–6]. No definite guidelines for MSCC treatment are available. In general, palliative decompressive surgery could be considered in cases where progressive neurologic deficit is observed, physical activity is relatively good, and multiple metastasis has been proven. Moreover, the need for surgical treatment is increasing, as longer associated survival is expected and the surgical technique has been well studied [7].

Palliative decompression surgery for MSCC is associated with problems such as potential massive bleeding and postoperative complications [8]. Several authors have suggested that preoperative embolization could reduce intraoperative blood loss in cases of hypervascular tumors and could thus simplify the operative procedures [9–11]. However, some studies have indicated that the blood loss does not differ when embolization is performed in cases of non-hypervascular tumors [12, 13]. Moreover, the risk of cord infarction or progression of the neurologic status as a result of operative delay are concerns related to the preoperative embolization procedures. Hence, the clinical benefits and risks of preoperative embolization need to be carefully evaluated, particularly in non-hypervascular tumors. To our knowledge, no definitive conclusion or guideline has been established with regard to the clinical importance of preoperative embolization.

In the present study, we aimed to compare the perioperative clinical outcomes based on whether preoperative embolization is performed in patients with MSCC and to assess the role and safety of preoperative embolization.

## Methods

### Patients and clinical parameters

We enrolled a total of 52 consecutive patients (37 men and 15 women) who underwent palliative decompression for MSCC from March 2012 to December 2014 at a single center. Demographic data, neurologic status, surgery-related data (operation time, estimated blood loss, and transfusion), complications, and survival time were recorded from the electronic medical records at our institution. The neurologic status was assessed based on the motor strength of the lower extremity: grade 0, no muscle contraction; grade 1, minimal muscle contraction; grade 2, active movement under gravity-free conditions; grade 3, active movement under gravity; grade 4, active movement under limited resistance; and grade 5, no weakness. If the values differed between the right and left lower extremities, the average values were adopted for analysis. The performance status was assessed using the Karnofsky performance status scale. The degree of tumor involvement was assessed by Bilsky scale [14]. The degree of instability was evaluated by Spinal Instability Neoplastic Score (SINS) [15]. Operation time was measured from skin incision to closure. Intraoperative blood loss was estimated based on the difference in the volume of suction drainage and irrigation fluid. The degree of transfusion was determined according to the use of packed red blood cells (RBC). The complications included surgical (hematoma, infection, or wound dehiscence) and medical (pneumonia, cardiac problem, or sudden death) problems. Although angiography was performed to assess hypervascularity as far as possible, we did not perform angiography because of the following reasons in many cases: (1) rapid progression of neurologic deficit, (2) lack of support such as absence of interventional radiologists, and (3) surgeon’s judgment after consideration of risk and benefit of embolization. Patients were categorized based on whether they underwent preoperative embolization: group E (embolization) and group NE (non-embolization). The clinical parameters were compared between the two groups. Subgroup analysis was performed specifically in the cases of hypervascular tumors (hepatocellular carcinoma, renal cell carcinoma, and thyroid cancer).

### Embolization procedure

**Fig. 1:**
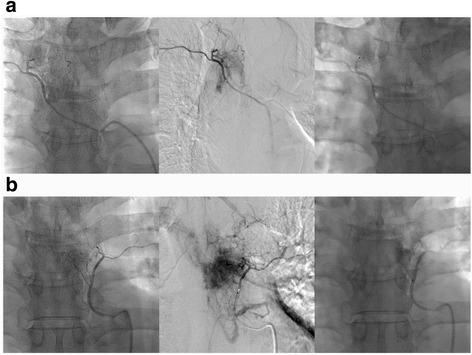
Angiography and embolization procedure in a 52-year old HCC patient with MSCC at T6. **a** Angiogram at the left 6th intercostal artery was obtained (*left*), hypervascular staining was observed (*middle*), and embolization was successful (*right*). **b** The same procedure was repeated at the right 6th intercostal artery. Hepatocellular carcinoma (HCC), metastatic spinal cord compression (MSCC)

Embolization was performed by radiologists with the patient under local anesthesia. A 4-F or 5-F catheter was used to perform diagnostic angiography. Angiography of the vertebral arteries, segmental arteries, and feeding branches supplying the tumors was performed. Thereafter, embolization with polyvinyl alcohol (PVA) particles and/or gelatin sponge (Gelfoam) was performed. If the branches supplying the tumors could be approached directly, then the particles were infused by introducing a microcatheter. If these branches could not be approached, then the normal branches distal to the feeder were protected by Gelfoam or coil prior to the infusion of particles. If the feeding vessels were too small to be identified, embolization was not performed. Repeat angiography was performed immediately to confirm residual tumor staining after embolization. An example of the embolization procedure is shown in Fig. 1.

### Surgical procedure

**Fig. 2:**
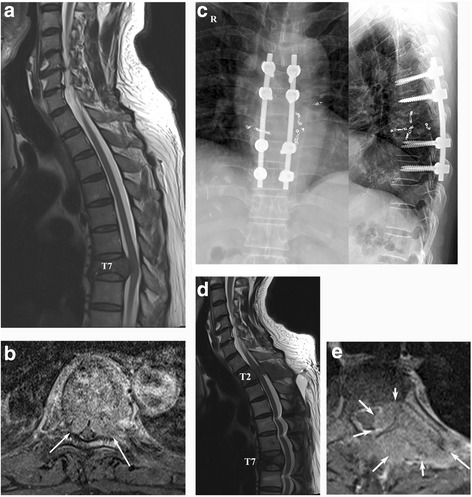
A 54-year old man with HCC who developed MSCC. **a** Preoperative T2-weighted sagittal MR image showing cord compression at *T7*. **b** Preoperative T1-weighted enhanced MR image (*arrows* indicate cord compression by the tumor mass). **c** Postoperative radiographs. **d** T2-weighted sagittal MR image 6 months postoperatively showing another occurrence of cord compression at *T2*, with maintenance of the decompression at *T7*. **e** T1-weighted enhanced MR image at *T2* (*arrows* indicate tumor mass). Hepatocellular carcinoma (HCC), metastatic spinal cord compression (MSCC)

Surgery was performed within 48 h after embolization. Instrumentation was performed using pedicle screws and rods across two levels, above and below the lesion. Thereafter, posterior decompression with total laminectomy was performed. Additional tumor resection was performed in certain cases. However, corpectomy of the vertebral body via the transpedicular approach or anterior approach was not performed. Bone grafting was also not routinely performed because of the short-term life expectancy of the patients. Two suction drains were placed after careful hemostasis. An example of a surgical case in a patient with MSCC is shown in Fig. 2.

### Statistical analyses

The demographic and operation-related parameters were compared between the two groups by using the independent *t* test, Mann Whitney *U* test, chi-square test, or Fisher’s exact test. Survival analysis was conducted with the Kaplan-Meier method and log-rank test. All statistical analyses were performed using the Statistical Package for the Social Sciences version 21.0 (SPSS Inc., Chicago, IL); *P* values <0.05 were considered to be statistically significant.

## Results

### Demographic data

The mean patient age at the time of operation was 59.7 ± 12.3 years. The most common origin of the cancers was hepatocellular carcinoma (HCC) (*n* = 12, 23.1%), followed by renal cell carcinoma (RCC) (*n* = 10, 19.2%) and lung cancer (*n* = 9, 17.3%). The most commonly involved sites were T6 (*n* = 9) and T7 (*n* = 10), followed by T3 (*n* = 8) and T4 (*n* = 7). The estimated blood loss was 1.22 ± 0.95 L, and the mean degree of transfusion was 3.9 ± 4.2 pints. The operation time was 198 ± 54 min.

### Preoperative embolization

Of the 52 study patients, 24 (46.2%) underwent preoperative angiography. However, 6 patients did not undergo embolization due to the presence of small arteries (*n* = 3), risk of cord infarction (*n* = 2), and difficulty with accessibility due to the presence of markedly tortuous vessels (*n* = 1). Thus, 18 patients (34.6%) underwent preoperative embolization. The involved vessels were segmental arteries or intercostal arteries that supply the tumors. No procedure-related complications were observed in 18 cases. Complete or near-complete (>80%) embolization was possible in 13 cases (72.2%). Partial embolization was performed in the other 5 cases due to the risk of cord ischemia or an inaccessible location. The time period between embolization and the surgical procedure was 16.4±9.1 h.

### Comparisons of demographic and operation-related parameters

Eighteen and 34 patients were assigned to groups E and NE, respectively. The preoperative demographic data did not significantly differ between the two groups (Table 1). However, the neurologic status in the NE group was inferior to that in the E group (2.7 vs 3.6), although the difference was not significant (*P* = 0.067). The postoperative neurologic status was also different between the two groups (2.9 vs 3.8, *P* = 0.042); however, the degree of neurologic improvement did not significantly differ (*P* = 0.519). Moreover, the degree of transfusion was greater in the NE group (4.6 pints) than in the E group (2.5 pints, *P* = 0.025). Blood loss, operation time, and complications did not differ between the two groups. However, massive bleeding (>2000 mL) was more frequent in the NE group (10/34) than in the E group (0/18, *P* = 0.010). A comparison of the intraoperative and perioperative parameters is described in Table 2.

**Table 1:** Demographic data of two groups classified by preoperative embolization

	Group NE (*n* = 34)	Group E (*n* = 18)	*P* value
Age (year)	60.7 ± 12.6	57.7 ± 11.6	0.396
Sex	M:F = 21:13	M:F = 16:2	0.055
Hypervascular tumor	13/34 (38.2%)	10/18 (55.6%)	0.232
Preop. RTx	11/34 (32.4%)	7/18 (38.9%)	0.637
Bilsky scale (Gr1:Gr2:Gr3)	6:11:17	2:7:9	0.790
Preop. neurologic status (Gr)	2.7 ± 1.7	3.6 ± 1.4	0.058
SINS	8.2 ± 2.1	7.2 ± 1.8	0.070
Site (≥T5:<T5)	14:20	7:11	0.873
Karnofsky performance	64.1 ± 14.0	71.7 ± 12.0	0.058

Means and standard variation are shown for continuous variables, and the number of cases is shown for categorical variables

*NE* non-embolization, *E* embolization, *Preop* preoperative, *RTx* radiotherapy, *SINS* Spinal Instability Neoplastic Score, *Gr* grade

**Table 2:** Intraoperative and perioperative parameters between two groups

	Group NE (*n* = 34)	Group E (*n* = 18)	*P* value
Postop. neurologic status (Gr)	2.9 ± 1.8	3.8 ± 1.3	0.066
Postop. neurologic improvement	12/34 (35.3%)	8/18 (44.4%)	0.519
Op. time (min)	197.0 ± 60.1	201.2 ± 42.1	0.790
Levels of laminectomy	1.3 ± 0.5	1.2 ± 0.5	0.604
EBL (L)	1.37 ± 1.11	0.99 ± 0.47	0.098
Transfusion (pint)	4.6 ± 4.9	2.5 ± 1.5	0.025
Massive bleeding	10/34 (29.4%)	0/18 (0%)	0.010
Complication	9/34 (26.5%)	5/18 (27.8%)	0.919

Massive bleeding is defined by intraoperative bleeding more than 2 L

*NE* non-embolization, *E* embolization, *Postop.* postoperative, *Op*. operation, *EBL* estimated blood loss, *Gr* grade

Of the 52 patients, 23 (44%) exhibited hypervascular tumors. A greater amount of intraoperative blood loss was observed in the cases with hypervascular tumors (1600 mL vs 916 mL, *P* = 0.015). Although massive bleeding was more frequently noted in cases with hypervascular tumors, 3 patients with non-hypervascularized tumors (prostate cancer, germ cell tumor, and breast cancer) also showed massive bleeding (*P* = 0.087). The comparisons between hypervascular and non-hypervascular tumors are described in Table 3.

**Table 3:** Comparisons between hypervascular and non-hypervascular tumor

	Hypervascular (*n* = 23)	Non-hypervascular (*n* = 29)	*P* value
Age	55.8 ± 10.3	62.7 ± 13.0	0.043
Sex	M:F = 19:4	M:F = 18:11	0.132
Preop. neurologic status (Gr)	3.1 ± 1.7	2.9 ± 1.6	0.571
Postop. neurologic status (Gr)	3.2 ± 1.7	3.3 ± 1.6	0.857
Bilsky scale (Gr1:Gr2:Gr3)	5:6:12	3:12:14	0.370
SINS	7.7 ± 1.9	8.0 ± 2.1	0.505
Karnofsky performance	67.8 ± 14.8	65.9 ± 13.0	0.612
Number of laminectomy	1.2 ± 0.5	1.3 ± 0.5	0.553
Op. time (min)	204.6 ± 61.7	193.4 ± 47.4	0.466
EBL (L)	1.60 ± 1.14	0.92 ± 0.64	0.015
Transfusion (pint)	4.5 ± 4.4	3.4 ± 3.9	0.366
Massive bleeding	7/23 (30.4%)	3/29 (10.3%)	0.087
Complication	4/23 (17.4%)	10/29 (34.5%)	0.217

*Gr* grade, *EBL* estimated blood loss, *SINS* Spinal Instability Neoplastic Score

Subgroup analysis indicated that intraoperative blood loss was greater in the NE group (1988 mL) than in the E group (1095 mL, *P* = 0.042) in cases of hypervascular tumors. Although 53.8% of patients in the NE group exhibited massive bleeding, none of the patients in the E group exhibited massive bleeding (*P* = 0.007). The results of subgroup analysis for cases of hypervascular tumors are summarized in Table 4. Subgroup analysis between HCC (12 cases) and RCC (10 cases) showed no differences in demographic data, operation-related data, and clinical outcomes. In addition, no differences according to involved levels (T1–5 vs T6–12) could be found.

**Table 4:** Subgroup analysis for hypervascular tumors (HCC, RCC, and thyroid ca.)

	Group NE (*n* = 13)	Group E (*n* = 10)	*P* value
Preop. neurologic status (Gr)	2.4 ± 1.7	4.0 ± 1.4	0.023
Postop. neurologic status (Gr)	2.4 ± 1.8	4.2 ± 1.0	0.006
Postop. neurologic improvement	4/13 (30.8%)	4/10 (40.0%)	0.685
Op. time (min)	208.3 ± 78.8	199.8 ± 31.3	0.790
EBL (L)	1.99 ± 1.37	1.10 ± 0.40	0.042
Transfusion (pint)	5.6 ± 5.6	3.0 ± 1.5	0.156
Massive bleeding	7/13 (53.8%)	0/10 (0%)	0.007
Complication	2/13 (15.4%)	2/10 (20.0%)	1.000

*Gr* grade, *EBL* estimated blood loss

### Perioperative complications and survival analysis

The most common complications of palliative decompression for MSCC were pulmonary problems (7/52, 13.5%) and wound problems (6/52, 11.5%) including seroma formation. Four patients (7.7%) exhibited wound dehiscence, and repeated debridement and advancement flap operations were performed by plastic surgeons. Another 2 patients showed postoperative hematoma; 1 patient was successfully treated via hematoma evacuation, whereas the other showed permanent neurologic deficits following hematoma evacuation. In that case, massive bleeding was observed, and blood loss persisted after surgery due to the reduced coagulative ability. After hematoma evacuation, angiography and embolization were performed to control the bleeding. The perioperative complications in the overall cohort are summarized in Table 5.

**Table 5:** Summary of perioperative complications

Case	Origin	Group	EBL	Complications	Progression
1	Lung cancer	NE	1500	Respiratory failure	Death in 1 week
2	Lymphoma	NE	1500	Wound dehiscence	Advancement flap by plastic surgeon
9	Breast cancer	NE	2000	Hematoma	Neurologic recovery after hematoma evacuation
10	RCC	NE	3000	Pneumonia	Recovery after medical treatment
14	Plasmacytoma	NE	1000	Pulmonary thromboembolism	Recovery after insertion of IVC filter, anticoagulation therapy
21	RCC	E	1600	Pneumothorax	Tracheostomy status. Expire in 3 months due to respiratory failure
25	Rectal cancer	E	300	Wound dehiscence in 1 month	Advancement flap by plastic surgeon
35	RCC	E	800	Wound dehiscence	Advancement flap by plastic surgeon
38	Esophageal cancer	NE	400	Pneumonia	Recovery after medical treatment
40	Breast cancer	NE	400	Dural tear, seroma	Observation
43	Lung cancer	E	600	Wound infection in 1 month	Debridement (+). Death in 6 weeks due to respiratory failure
50	Klatskin tumor	E	400	Dural tear, wound dehiscence	Advancement flap by plastic surgeon
51	myeloma	NE	200	Atelectasis	Recovery after chest tube insertion
52	HCC	NE	4000	Neurologic deficit by hematoma, respiratory failure	Persistent neurologic deficit after hematoma evacuation

*EBL* estimated blood loss, *RCC* renal cell carcinoma, *HCC* hepatocellular carcinoma, *E* embolization, *NE* non-embolization, *PS* plastic surgery

**Fig. 3:**
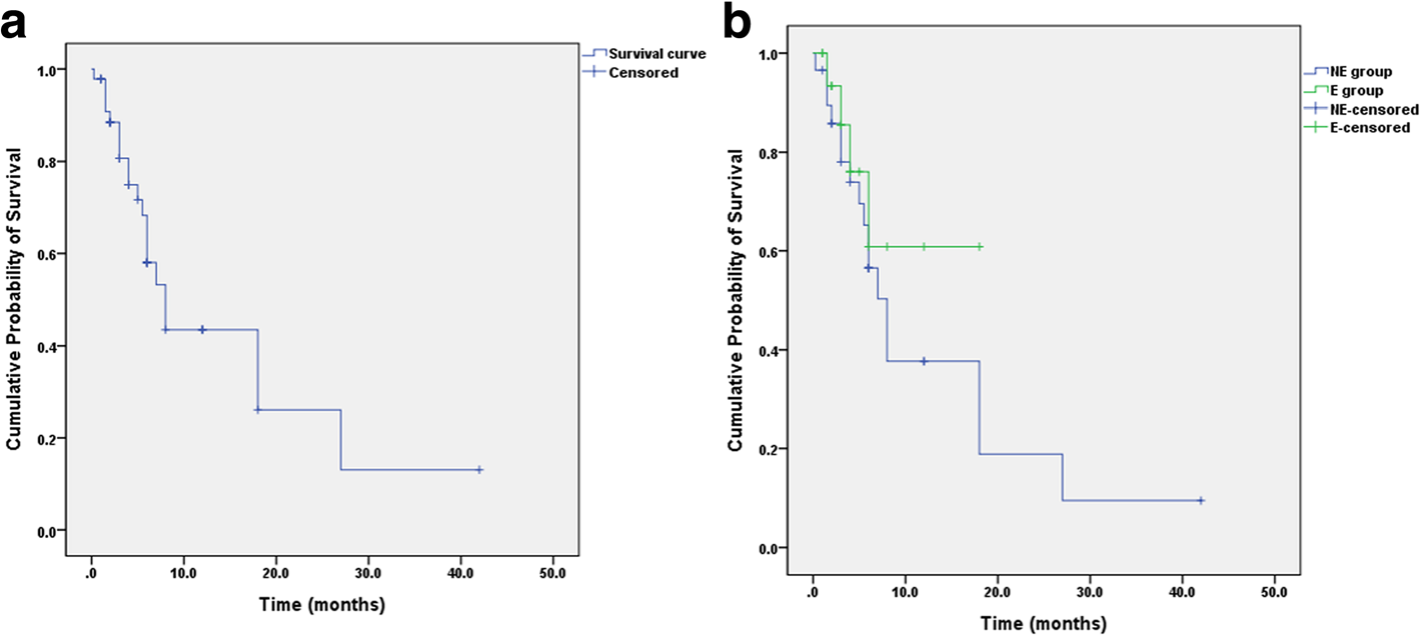
Survival analysis. **a** Kaplan-Meier survival curve for all patients. **b** Comparison of the survival curve according to whether preoperative embolization was performed

The mean estimated survival time was 14.8 ± 5.9 months, and the median survival time was 8.0 ± 2.6 months. The Kaplan-Meier survival curve is illustrated in Fig. 3. Survival did not significantly differ between the NE group and E group (*P* = 0.321).

## Discussion

Patients with MSCC are frequently encountered in the clinical setting. Moreover, as most metastatic spinal tumors are hypervascular in nature, it is essential to focus on decreasing the intraoperative blood loss [16]. Several reports have described the usefulness of preoperative embolization in reducing intraoperative blood loss in spinal tumors [10, 17, 18]. In particular, embolization has been reported to be effective for reducing blood loss in hypervascular tumors such as RCC [9, 12, 18]. However, several reports have suggested that the procedure does not decrease blood loss, and instead, care must be taken to avoid cord ischemia [19]. Furthermore, it was reported that there was no difference in blood loss between the use of local hemostatic agents and preoperative embolization [20]. Thus, no definitive guidelines regarding preoperative embolization have been established for the treatment of MSCC patients.

To determine the superiority of the routine clinical use of preoperative embolization, the assessment of the risk of the procedure is critical. In our present study, none of the patients showed procedure-related complications, including neurologic deficits. However, embolization was not performed in 2 patients because of the risk of cord infarction due to occlusion of the anterior spinal arteries. In fact, cord ischemia may be the most critical complication of this procedure. Based on an animal study, contiguous ligation of three segmental vessels bilaterally did not lead to any neurologic compromise [21]. However, the ligation of >4 levels produced ischemic cord dysfunction in a dog model [22]. Therefore, we performed embolization up to two levels bilaterally, and up to three levels unilaterally, which we considered to be safe. The safety of embolization has also been reported by several authors [10, 23, 24]. However, most of these authors also indicated the risk of cord ischemia.

The factors associated with the effectiveness of preoperative embolization remain unclear. Although it could be reasonable that less blood loss was found in cases of complete occlusion [11, 25], no difference in blood loss was observed according to the completeness of the occlusion [17, 23]. In addition, the time interval may be another important parameter. Some authors proposed that preoperative embolization was effective when the time from the procedure to the index surgery was <24 h [25]. In contrast, other authors did not find any correlation between the time interval and intraoperative blood loss [17]. Furthermore, as surgical timing is critical for mitigating a decline in neurologic function, a delay in the operation due to preoperative embolization may be a problem. However, in the present study, we found that none of the patients showed a progression of neurologic deficits, even though the average time interval between the procedure and the index operation was 16 h. Nevertheless, this finding was not conclusive, as patients with more severe neurologic deficit tended to undergo surgery more promptly without embolization (*P* = 0.067).

The abovementioned controversial results could be attributed to the retrospective nature of those studies. A randomized controlled trial showed no difference in blood loss and transfusion based on whether preoperative embolization was performed, although the patients with preoperative embolization did exhibit a reduction in surgical time [13]. However, that study had certain limitations, such as the heterogeneity of the population in terms of differing vascularity and a relatively small sample size. An assessment of the hypervascularity of the tumors would be critical for predicting intraoperative bleeding. However, no definite tools for such an assessment have been developed thus far. In particular, MRI characteristics are not known to be reliable for determining vascularity [26]. Therefore, we believe that routine angiography could be better for identifying tumor vascularity, as it is not associated with any adverse effects such as neurologic deterioration.

The present study had certain limitations of note. First, the number of patients who underwent preoperative embolization procedures was relatively small. Hence, the effect of complete or partial occlusion of feeder arteries could not be assessed. Second, some of the tumors classified as non-hypervascular tumors might have had some extent of hypervascularity. Previous reports have stated that breast cancer or prostate cancer occasionally exhibits hypervascular features [27]. Third, although surgery was performed by a single surgeon, angiography and embolization were conducted by multiple radiologists. Hence, the completeness of the embolization could be influenced by the technique or experience of the radiologist. Fourth, selection bias should be considered, as patients with greater neurological deterioration tended to be selected for emergent operation without embolization.

Despite these limitations, however, this study showed that preoperative embolization is relatively safe and effective for reducing intraoperative blood loss in surgery for MSCC. Furthermore, preoperative embolization is critical for minimizing the probability of massive bleeding in non-hypervascular as well as hypervascular tumors.

## Conclusions

In conclusion, intraoperative blood loss and perioperative transfusion can be reduced by using preoperative embolization in patients with MSCC. The neurologic recovery and complications do not differ based on whether embolization was performed. As preoperative embolization is relatively safe and effective for controlling intraoperative bleeding without any neurologic deterioration, it is highly recommended for hypervascular tumors. Furthermore, preoperative embolization can serve as a good option for non-hypervascular tumors, as massive bleeding was also found in certain cases.
